# A Foreground-Aware Framework for Local Face Attribute Transfer

**DOI:** 10.3390/e23050615

**Published:** 2021-05-16

**Authors:** Yuanbin Fu, Jiayi Ma, Xiaojie Guo

**Affiliations:** 1 College of Intelligence and Computing, Tianjin University, Tianjin 300350, China; yuanbinfu@tju.edu.cn; 2 Electronic Information School, Wuhan University, Wuhan 430072, China; jyma2010@gmail.com

**Keywords:** face attribute transfer, image warping, image fusion, facial landmark detection

## Abstract

In the context of social media, large amounts of headshot photos are taken everyday. Unfortunately, in addition to laborious editing and modification, creating a visually compelling photographic masterpiece for sharing requires advanced professional skills, which are difficult for ordinary Internet users. Though there are many algorithms automatically and globally transferring the style from one image to another, they fail to respect the semantics of the scene and are unable to allow users to merely transfer the attributes of one or two face organs in the foreground region leaving the background region unchanged. To overcome this problem, we developed a novel framework for semantically meaningful local face attribute transfer, which can flexibly transfer the local attribute of a face organ from the reference image to a semantically equivalent organ in the input image, while preserving the background. Our method involves warping the reference photo to match the shape, pose, location, and expression of the input image. The fusion of the warped reference image and input image is then taken as the initialized image for a neural style transfer algorithm. Our method achieves better performance in terms of inception score (3.81) and Fréchet inception distance (80.31), which is about 10% higher than those of competitors, indicating that our framework is capable of producing high-quality and photorealistic attribute transfer results. Both theoretical findings and experimental results are provided to demonstrate the efficacy of the proposed framework, reveal its superiority over other state-of-the-art alternatives.

## 1. Introduction

Sharing headshot photos taken by portable devices on networking sites is a new social form [1]. To produce a quality photographic work, laborious editing and modification of photos by well-trained photographers is needed. Unfortunately, most Internet users have not mastered the required professional skills, and one small mistake will ruin the whole work when editing a photo [2]. Hence, an automatic and interactive face photo editing algorithm is needed to avoid the unnecessary waste of time and effort. Ideally, if a reference headshot portrait is provided, say the target visual attribute that a user expects to obtain by editing their own face photo is clearly exhibited, we can intuitively and effortlessly transfer the visual attribute from a reference face photo to the photo taken by a user.

Recently, many neural-style transfer algorithms for globally transferring the artistic style from an image to another have been proposed [3,4,5,6,7,8,9,10]. However, existing global algorithms struggle to consider the semantics of the local face organs during the transfer process, for instance, the target attributes of eyes from the reference image may be wrongly transferred to hairs. Luan et al. [11] incorporated the semantic labeling of both the input image and the reference image into the style transfer algorithm to ensure that the transfer only occurs between two regions having the same semantic label. With the guidance of a semantic map, Luan et al. [11] can successfully avoided the spillover problem where the style of a region in the reference image spills over into a mismatched region in the input image. However, a fatal limitation of [11] is that it fails to solely render the semantic content in the foreground subregion instead of the whole image. In most cases, users may generally focus on the foreground, i.e., the human face in a headshot portrait, regardless of the appearance of the background; they only focus on if it is reasonable. More importantly, the background of headshot portraits is often quite diverse, leading to the background of the input and reference face photo not containing the semantically corresponding objects. For example, the background may be a seaside with sunshine and sand, skyscrapers in the city, or simply an all-white image. Directly copying the appearance of an object in the background of the reference image to a semantically unrelated object in the background of the input image will distort the spatial structures and cause painting-like effects. Therefore, it is important to develop an automatic algorithm to render the foreground region without affecting the background.

**Figure 1:**
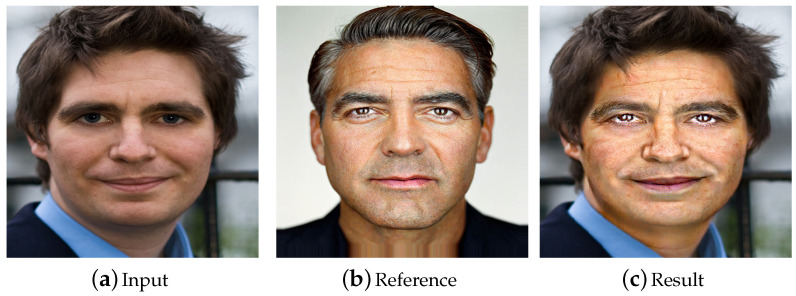
The attribute is transferred from a reference portrait (**b**) to an input image (**a**). Our technique is local and flexible, only altering the attribute of the foreground (in this case, the foreground regions include the eyes, mouth, and face skin) while maintaining the background unchanged. First, landmark detection is performed on the input and reference image. Then, the reference image is warped to match the input image. Finally, the fusion of the input image and warped reference image is taken as the initialization of the neural style transfer algorithm to create the output. Image courtesy of [2] (Link: https://people.csail.mit.edu/yichangshih/portrait_web/ accessed on 14 May 2021). Images are best viewed in color.

In this study, we aimed to build an efficient framework to allow users to simply edit the style of one or two face organs in the foreground while preserving the background. Figure 1 demonstrates that our framework is capable of ensuring that the attribute transfer only happens between semantically equivalent organs while the background remains unchanged. Here, we summarize the challenges in local face attribute transfer:

- Accurately and faithfully transferring the attribute from the reference headshot photography to the semantically-equivalent regions in the user’s own face photo;
- Avoid an unnatural and artificial transition between the foreground with the new attribute and the background with the original attribute to ensure the results resemble the photos directly taken by users, instead of a crude composition of some regions in the input and reference;
- Providing an interactive method for users to determine which regions are foreground and which are background.

To respect the semantics of local face organs, similar to [11], we introduce the semantic map of both the input and reference headshot photo to guide the attribute transfer process. Semantic channels and classes can be either annotated by users who aim to control the semantic correspondence between the input and reference, or learned by a face parsing and semantic segmentation network. We add a new channel/class, called background, to the semantic map, which corresponds to the region expected to remain unchanged. The semantic map is allowed to be annotated at a coarse level, as long as it is able to represent the semantically corresponding subregions between the input and reference images. However, it is still challenging for an algorithm to iteratively update a white-noise image to a desired result with local properties satisfied. Therefore, we propose a novel strategy to initialize the image, which involves warping the reference image to match the shape, pose, location, and expression of the input image using a thin plate spline. Our contributions can be summarized as follows:

- We introduce the semantic map of both the input and reference images for local face attribute transfer to produce a visually pleasing result using a semantically meaningful fashion. Equipped with the semantic map, we successfully achieve locally semantic-level attribute transfer (e.g., mouth-to-mouth), sufficiently improving the accuracy of the stylistic match.
- We add an additional background channel into our semantic map to indicate the background region required to be maintained the same as the input image. We also provide an effective initialization strategy and propose a novel term, a *preservation term*, to flexibly handle the particular demand that merely manipulates the attribute of the foreground region, while preserving the background region.
- We conduct extensive experiments to reveal the efficacy of our design, and demonstrate its advantages over other state-of-the-art methods.

## 2. State of the Art

### 2.1. Global Neural Style Transfer

Image style transfer is a research hotspot with considerable commercial potential, and has been widely studied by industry and academia. In the past four to five years, researchers have proposed a variety of style-transfer methods, which can be divided into traditional methods and deep-learning methods. This section introduces the representative works of these two categories.

For a long time, many impressive artworks painted by famous artists have inspired computer vision researchers to explore how to automatically create an appealing masterpiece. The earliest attempt arguably traces back to non-photorealistic rendering [12,13], in which the main limitation is the design for a particular style. Recently, Ref. [14] defined a new problem called image analogies to synthesize a new $B^{\prime }$ from *B* according to the given pair $A^{\prime }$ and *A*, which allows users to simply provide an exemplar and produce a synthesis result similar to it. Men et al. [15] proposed a common texture transfer framework that regards texture transfer as an image inpainting problem, and produces the target image according to the original image and the semantic map. Specifically, this method first warps the original image according to the semantic map of the original image and target image to obtain the prior target image, then inpaints the warped image using PatchMatch to obtain the final result. However, the weakness of these two methods is that they solely use low-level features to inform the style transfer process.

In addition to the above-mentioned traditional approaches, since Gatys et al. [3] proposed using CNNs for neural style transfer, researchers have increasingly focused on this research field, and a large number of studies using deep learning for style transfer is published every year [1], which has boosted the performance of neural style transfer. As the pioneering work of this category, the algorithm proposed by [3] uses pre-trained VGG16 [16] to extract the content and style features, and iteratively updates an image randomly initialized by white noise to minimize the content and style losses. Johnson et al. [4] further proposed a novel network architecture consisting of a transform network and a loss network to accelerate the processing speed. The loss network is frozen during training, and the transform network is trained using a style image and multiple content images. In the testing phase, only a feed-forward process is needed. Stylebank [5] introduces the StyleBank layer between the encoder and decoder. The StyleBank layer contains multiple FilterBanks, and a FilterBank corresponds to a style, which helps to handle multiple styles in one model. Shen et al. [9] developed an algorithm for training a meta network to generate the parameters of another transform network for arbitrary style transfer. In the testing phase, one can simply take an arbitrary style image as the input to the meta network to obtain the parameters of the transform network, then the transform network processes a content image using the generated parameters to produce the final style transfer result. Gu et al. [17] designed a novel loss term to combine the advantages of both parametric and non-parametric methods. Gu et al.’s [17] method can preserve the richness of style rendering, and it can improve the faithfulness of the stylistic match. Huang et al. [10] attempted to perform neural style transfer for videos. To constrain the consistency between different frames, in addition to the content and style loss on each frame, the method [10] predicts the frame at time *t* in a video from the frame at t−1 using optical flow, and designs a new temporal loss to calculate the Euclidean distance between the ground truth and the predicted frame at time *t* in a video.

Though achieving visually pleasing results, the above-mentioned neural style transfer approaches ignore the semantics of the scene due to their global nature, and are unable to transfer specific styles like makeup style [18], face style [19], photography style [2], and comic style [20].

### 2.2. Face Attribute Manipulation

Face attribute manipulation is the process of editing face attributes such as age, lighting, expression, and identity. Early works of face attribute manipulation were carefully designed for one particular attribute. For example, the work of [21] overcame the difficulty where adequate aligned data are lacking for the same person at different ages, and managed to take a photo of a little child as the input and generated multiple results at different ages by properly altering the pose, expression, and illumination. Blanz et al. [22] proposed an approach for processing the face shown in an image or a video, which does not heavily depend on the data of different person’s attributes. SHBMM [23] integrates spherical harmonics into a morphable model framework to represent a face under arbitrary lighting conditions using three low-dimensional vectors (shape parameters, spherical harmonic basis parameters, and illumination coefficients); even the geometry and albedo of the face are unknown, so the method is robust not only to extreme lighting conditions, but also to partial occlusions. Yang et al. [24] corrected an undesirable expression in a face photo by transferring the facial expression from another image, similar to [25]. To avoid semantically unnatural composites, Ref. [24] presents a 2D flow field to naturally warp the target face by projecting the constructed 3D shapes back to 2D.

In comparison with the recent development of generative adversarial networks, different facial attributes are allowed to be handled by changing the training data. Typically, facial attribute manipulation can be regarded as an image-to-image translation problem, which aims at mapping images from a source domain to a target domain. Different facial attributes belong to different domains. An early attempt at image-to-image translation by Isola et al. [26] uses conditional GANs to learn mappings between the two domains. The content preservation is supervised by the paired data. However, in real-world situations, acquiring paired datasets is time consuming and laborious. To alleviate this problem, inspired by the concept of cycle consistency, cycleGAN [27], DualGAN [28], and DiscoGAN [29] can be trained without paired datasets. Afterward, many works [19,30,31,32] further extended the translation between the two domains to cross multiple domains in a single model. Though the effectiveness of these GAN-based approaches has been verified by various applications, their main drawback is their instability in training and the difficulty of interpretation.

Deep feature interpolation [33] is able to avoid the limitations of generative adversarial nets, which alters the latent code of a face image learned by a shallow CNN to update its attributes. Cong et al. [34] further alleviated the problems in [33] of the noisy estimation of latent code and the high computational burden. Inspired by CapsuleNet [35], they parsed a face image into multiple smaller parts to divide a high-level attribute, such as expression, age, or sex, into multiple semantic components. The main weakness of these two deep feature interpolation methods is that the numerical latent code is not intuitive, and it does not contain the visual and spatial information of the attribute. In other words, the numerical latent code is unable to spatially reflect the visual look or appearance of each subregion in the input image.

Different from the above-mentioned face attribute manipulation approaches, we aimed to transfer the visual attribute from one face image to another without needing to explicitly define the type of attribute such as the expression, age, or identity. In other words, users can simply copy the visual appearance of a reference image, such as the color, texture, and style, to another image, to ensure the processed result is similar to the reference, which is a more intuitive and straightforward method compared with numerical latent code. Our framework is flexible enough to handle several different face images and does not require a time-consuming and unstable training procedure.

## 3. Methodology

**Figure 2:**
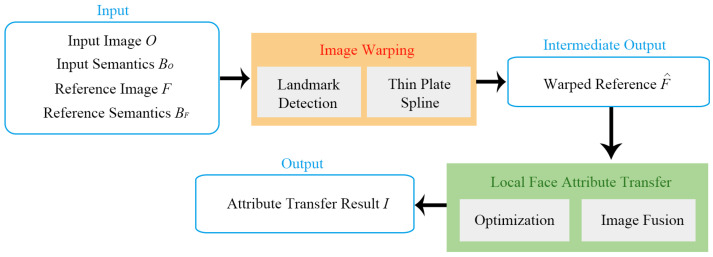
The outline of our framework.

Our goal was to transfer the visual attribute from the reference headshot photo *F* to the input photo *O* guided by the semantic map $B_{F}$ and $B_{O}$ of the reference and input, respectively. The identity of a person should be maintained during the attribute transfer process. We start by obtaining the facial landmark of both the input and reference, the advantage of which is that the facial landmark points of two different face images are already registered, without needing to establish a dense correspondence between them using an off-the-shelf point registration method. We then warp the reference photo using a thin plate spline according to the detected facial landmark points to match the shape, pose, and position of the input photo. The warped reference photo is then fused with the input photo. Finally, the fusion image is taken as the initialized image of the neural style transfer algorithm with our newly proposed preservation term as the objective to be optimized to produce the final face attribute transfer result. The blueprint of our framework is schematically illustrated in Figure 2.

### 3.1. Facial Landmark Detection

**Figure 3:**
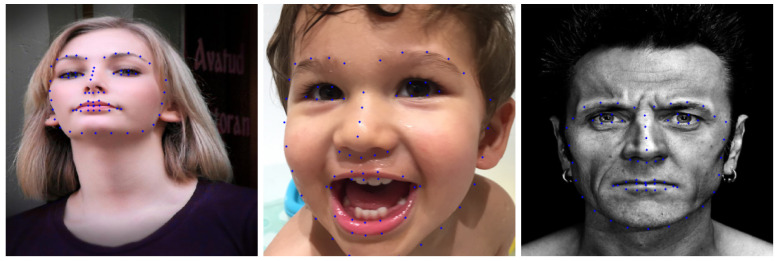
Some examples of facial landmark detection. The total number of landmark points detected by RCT [42] is 68.

Facial landmark detection involves marking the vital parts of a human face with key points in the face image, which has been widely used in pose estimation [36,37], face alignment [38], expression recognition [39,40], and face location [41]. Facial landmarks contain rich semantic information such as human eyes, nose, hair, and mouth, which can help a neural network to transfer facial attributes between semantically equivalent subregions. In addition, using the same model to detect different face images, the registered landmark points can be obtained, which can be directly used as the key points of image warping, without needing to apply other point registration algorithms to establish a dense correspondence between the key points of different images. Based on the above reasons, we adopted the pre-trained RCT model [42] to detect facial landmarks, which detects a total of 68 points as the prior information for later process steps. Some facial landmark detection results of the RCT model are shown in Figure 3.

### 3.2. Thin Plate Spline

**Figure 4:**
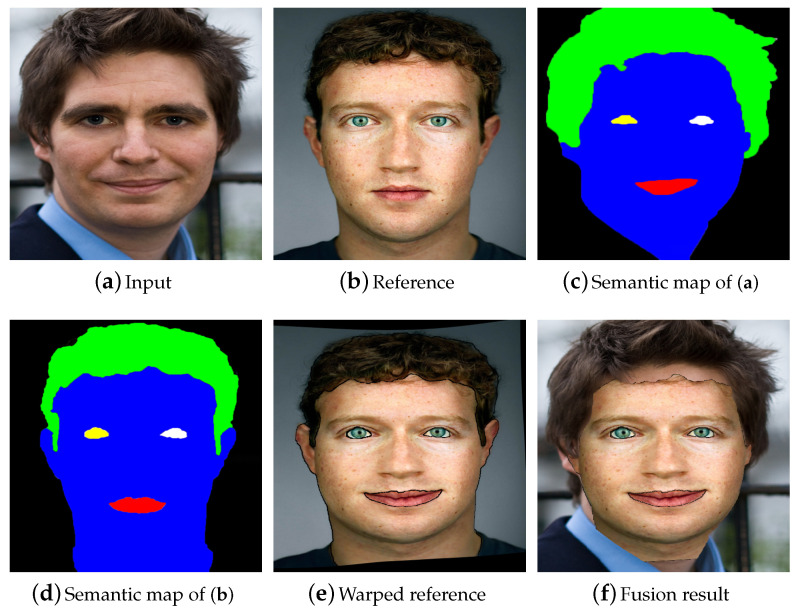
The results of image warping and fusion. (**c**,**d**) The semantic maps of (**a**,**b**), respectively. Left eye, right eye, mouth, face skin, hair, and background are labeled in yellow, white, red, blue, green, and black in the semantic map, respectively. (**e**) The result of warping (**b**) to match (**a**) by thin plate spline; (**f**) the image fusion result. In this case, hair is also classified as the background to be maintained.

Thin plate spline, proposed by [43], is a non rigid image warping technology. In this method, given some registered control points in two images, one image is warped so that its control points coincide with the control points of the other image. An example of image warping by thin plate spline is shown in Figure 4. For the input photo and the reference headshot portrait with different shapes, poses, positions, and facial expressions, thin plate spline can be used to warp the reference face photo to match the input photo with the facial key points provided.

Let $L_{O}:={\left[o_{1},o_{2},\ldots ,o_{N}\right]}^{T}\in R^{N\times 2}$ and $L_{F}:={\left[f_{1},f_{2},\ldots ,f_{N}\right]}^{T}\in R^{N\times 2}$ denote the 2D coordinates of the facial landmark points detected from the input image and reference image, respectively. Thin plate spline can then be expressed as:

(1) $$\begin{array}{cc} o_{k}:=\Phi \left(f_{k}\right) & :={\left[\Phi_{x}\left(f_{k}\right),\Phi_{y}\left(f_{k}\right)\right]}^{T}\\ \mathrm{with}\;\;\Phi_{x}\left(f_{k}\right) & :=a_{x}+b_{x}^{T}f_{k}+\omega_{x}^{T}S\left(f_{k}\right)\\ \Phi_{y}\left(f_{k}\right) & :=a_{y}+b_{y}^{T}f_{k}+\omega_{y}^{T}S\left(f_{k}\right) \end{array}$$

where N=68 is the total number of facial landmark points detected by RCT [42]; $\Phi_{x}\left(•\right)$ and $\Phi_{y}\left(•\right)$ represent the interpolation function with respect to the x-axis and y-axis in two-dimension coordinates, respectively; and $S\left(f_{k}\right)\in R^{N\times 1}$ is a column vector used to calculate the distance between $f_{k}$ and the other *N* landmark points of an image. The element of $S(f_{k})$ in row *i* is $\sigma (||f_{k}-f_{i}|{|}_{1})$; σ is the radial basis function whose formulation is $\sigma \left(r\right):=r^{2}\log r$.

To solve the exact solution of Φ(•), we need to separately solve the parameters of $\Phi_{x}\left(•\right)$ and $\Phi_{y}\left(•\right)$. Taking $\Phi_{x}\left(•\right)$ as an example, because the number of parameters to be solved is N+3, as the number of given measurements is *N*, we need to introduce three extra constraints to solve $a_{x}\in R^{1\times 1}$, $b_{x}\in R^{2\times 1}$, and $\omega_{x}\in R^{N\times 1}$ in $\Phi_{x}\left(•\right)$. The three additional constraints are:

(2) $$\begin{array}{cc} \sum_{k=1}^{N}\omega_{x,k} & =0\\ \sum_{k=1}^{N}\omega_{x,k}f_{k}^{x} & =0\\ \sum_{k=1}^{N}\omega_{x,k}f_{k}^{y} & =0 \end{array}$$

where $\omega_{x,k}$ denotes the *k*th element of the column vector $\omega_{x}$; $f_{k}\in R^{2\times 1}$ represents the 2D coordinates of the *k*th facial landmark point detected from the input image; $f_{k}^{x}$ and $f_{k}^{y}$ denote the x-coordinate and y-coordinate of the *k*th landmark point, respectively. The formulation of $\Phi_{y}\left(•\right)$ is similar to $\Phi_{x}\left(•\right)$, so we do not repeat it again.

After introducing the above three constraints, the parameters of $\Phi_{x}\left(•\right)$ and $\Phi_{y}\left(•\right)$ can be determined by solving the following equation:

(3) $$\left[\begin{array}{ccc} S(L_{F}) & 1_{N} & L_{F}\\ 1_{N}^{T} & 0 & 0\\ L_{F}^{T} & 0 & 0 \end{array}\right]\left[\begin{array}{cc} \omega_{x} & \omega_{y}\\ a_{x} & a_{y}\\ b_{x} & b_{y} \end{array}\right]=\left[\begin{array}{cc} L_{O}^{x} & L_{O}^{y}\\ 0 & 0\\ 0 & 0 \end{array}\right]$$

where $L_{O}^{x}\in R^{N\times 1}$ and $L_{O}^{y}\in R^{N\times 1}$ denote the x-axis and y-axis of the facial landmark points detected from the input image, respectively; $1_{N}\in R^{N\times 1}$ is an all-one vector column; and $S\left(L_{F}\right)\in R^{N\times N}$ represents the distance between any two facial landmark points. The element of $S(L_{F})$ in the *i*th row and *j*th column is $\sigma (||f_{i}-f_{j}|{|}_{1})$.

When the optimal parameters of Φ(•) are solved, which are $a_{x}^{*}$, $b_{x}^{*}$, and $\omega_{x}^{*}$ for the x-axis; and $a_{y}^{*}$, $b_{y}^{*}$, and $\omega_{y}^{*}$ for y-axis, we need to calculate the distance between each pixel in the reference image and its all landmark points by the radial basis function to obtain $S\left(P_{F}\right)\in R^{M\times N}$, where $P_{F}\in R^{M\times 2}$ is the coordinates of all pixels in the reference image and *M* is the number of pixels. The new coordinates of the pixels in the warped reference image are therefore obtained by:

(4) $$\left[\begin{array}{cc} P_{\hat{F}}^{x} & P_{\hat{F}}^{y}\\ 0 & 0\\ 0 & 0 \end{array}\right]:=\left[\begin{array}{ccc} S(P_{F}) & 1_{M} & P_{F}\\ 1_{M}^{T} & 0 & 0\\ P_{F}^{T} & 0 & 0 \end{array}\right]\left[\begin{array}{cc} \omega_{x}^{*} & \omega_{y}^{*}\\ a_{x}^{*} & a_{y}^{*}\\ b_{x}^{*} & b_{y}^{*} \end{array}\right]$$

where $P_{\hat{F}}^{x}$ and $P_{\hat{F}}^{y}$ are the new x- and y-coordinate of all pixels in the warped reference image $\hat{F}$. We map each pixel in original reference image *F* to its new position represented by $P_{\hat{F}}:=\left[P_{\hat{F}}^{x},P_{\hat{F}}^{y}\right]:={\left[{\hat{f}}_{1},{\hat{f}}_{2},\ldots ,{\hat{f}}_{M}\right]}^{T}\in R^{M\times 2}$ to produce the warped reference image $\hat{F}$. If a new coordinate ${\hat{f}}_{k}$, corresponding to the pixel located at $f_{k}$ in the original reference image *F*, exceeds the range of the image size, the pixel located at $f_{k}$ in the warped reference image is set to zero if $\hat{f_{i}}\neq f_{k},\forall 1\leq i\leq M$.

### 3.3. Image Fusion

Having the warped reference image $\hat{F}$, we can initialize the image for neural style transfer by fusing the warped reference image with the input image, instead of randomly initializing the image by white noise. Specifically, the first step is to determine which facial organs need to be edited. The regions where attributes do not need to be changed are labeled as background in the semantic map, then the background regions in the input image *O* and the foreground regions in the warped reference image $\hat{F}$ are fused by the formulation described below:

(5) $$\begin{array}{c} U\left(i,j\right):=\left\{\begin{array}{cc} O(i,j) & O\left(i,j\right)\;\mathrm{is}\;\mathrm{background}\;||\hat{F}\left(i,j\right)=0,\\ \hat{F}\left(i,j\right) & \mathrm{else}. \end{array}\right. \end{array}$$

where *i* and *j* indicate the pixel at the *i*th row and *j*th column of an image, respectively; || denotes the *or* operation. We can infer from (5) that the pixel in the *i*th row and *j*th column in the input image is assigned to the pixel in same position in the fused image *U* if the corresponding label is background, or the pixel in same position in the warped reference image is zero. In other cases, the pixel in the warped reference image is assigned to the pixel in the same position in *U*. An example of image fusion is shown in Figure 4.

### 3.4. Optimization

We take the fused image *U* as the initialization of the image for the neural style transfer algorithm. Let I:=U denote the image for neural style transfer and, as in [3], we perform gradient descent on *I* to minimize the optimization objective. Our objectives consist of a content term, a style term, and a newly proposed term called the preservation term. The overall objective is formulated as:

(6) $$\begin{array}{c} L:=\lambda_{con}L_{con}+\lambda_{sty}L_{sty}+\lambda_{pre}L_{pre} \end{array}$$

where $\lambda_{con}$, $\lambda_{sty}$, and $\lambda_{pre}$ are the weights for balancing the different terms. During training, we alternatively optimize our preservation term, and the sum of the content and style terms.

The content term desires *I* and *O* to be as close as possible. We follow [3,4] to measure the distance between high-level features extracted from the pre-trained VGG16 instead of the pixel-wise reconstruction, the formulation of which is:

(7) $$\begin{array}{c} L_{con}:=\left|\right|\Psi_{\gamma }\left(I\right)-\Psi_{\gamma }\left(O\right){\left|\right|}_{2}^{2} \end{array}$$

where $\Psi_{\gamma }\left(•\right)\in R^{N_{\gamma }\times D_{\gamma }}$ refers to the vectorized feature map of a VGG layer, $N_{\gamma }$ is the number of feature maps, and $D_{\gamma }$ is the length of a vectorized feature map in the γth layer. With the content term, the identity of *I* is preserved, whereas other attributes such as texture and color are allowed to be changed.

The style term allows users to control the attributes by providing a reference photo. The original style term proposed by [3] is expressed as:

(8) $$\begin{array}{c} L_{sty}:=\sum_{\gamma }^{\Gamma }\left|\right|G_{\gamma }\left(I\right)-G_{\gamma }\left(F\right){\left|\right|}_{2}^{2} \end{array}$$

where $G_{\gamma }$ is the Gram matrix in the γth VGG layer, which is defined as $G_{\gamma }\left(•\right):=\Psi_{\gamma }\left(•\right)\Psi_{\gamma }{\left(•\right)}^{T}\in R^{N_{\gamma }\times N_{\gamma }}$.

However, most existing neural style transfer algorithms globally transfer the style to the whole input image, which ignores the semantics of each local object because the Gram matrix for the original style term is computed over the entire feature map. To mitigate this problem, inspired by [11], we introduce the semantic map as the additional information and reformulate the style term as:

(9) $$\begin{array}{c} L_{sty}:=\sum_{\gamma }^{\Gamma }\sum_{c=1}^{C}\left|\right|G_{\gamma ,c}\left(I\right)-G_{\gamma ,c}\left(F\right){\left|\right|}_{2}^{2} \end{array}$$

where $G_{\gamma ,c}\left(•\right):={\hat{\Psi }}_{\gamma ,c}\left(•\right){\hat{\Psi }}_{\gamma ,c}{\left(•\right)}^{T}$, ${\hat{\Psi }}_{\gamma ,c}\left(I\right):=B_{O,c}\circ \Psi_{\gamma }\left(I\right)$, and ${\hat{\Psi }}_{\gamma }\left(F\right):=B_{F,c}\circ \Psi_{\gamma }\left(F\right)$, ∘ denotes the Hadamard product; *C* is the total number of channels or classes in the semantic map, except for the background class; and *c* is the index of each channel or class in the foreground.

The preservation term is used to maintain the background region of *O* into style transfer result *I*. The formulation of th preservation term is:

(10) $$\begin{array}{c} L_{pre}:=||I\circ B_{O,g}-O\circ B_{O,g}{\left|\right|}_{1} \end{array}$$

where $B_{O,g}$ represents the background channel *g* in the semantic map. Different from the content term, our preservation term forces the pixels of output image *I* to exactly match the pixels of the input image *O*, since the background is not expected to be changed.

## 4. Experimental Validation

Our algorithm was implemented in PyTorch. All the experiments were carried out on a machine with a GeForce RTX 1060Ti GPU and an Intel Core i7-8700 3.20 GHZ CPU. The optimizer implemented the L-BFGS algorithm, and the images were all resized to 512 × 512 pixels. The weights were set to $\lambda_{con}:=3$, $\lambda_{sty}:=20$, and $\lambda_{pre}:=1$. Our code is available at https://github.com/ForawardStar/FaceAttributeTransfer accessed on 14 May 2021.

### 4.1. Evaluation Metrics

We evaluated our results using two quantitative metrics: inception score (IS) and Fréchet inception distance (FID).

#### 4.1.1. Inception Score

The inception score [44] is a widely-adopted metric for quantitatively measuring the visual quality of generated images, the name of which was inspired from the classification network proposed by [45], called InceptionNet. The inception score measures the generated images from two aspects: clarity and diversity. The principle behind the IS is that for a clear image *I*, the probability P(C|I) that *I* belongs to a specific class *C* should be very large, while the probability of belonging to other classes should be very small, since a clear image can be certainly and easily classified to a class but a blurred image cannot. If the patterns of the generated images are diverse, the probability distribution P(C) of all the classes should be uniform, which is different from the distribution of P(C|I). Inception score is therefore the KL divergence of P(C|I) and P(C). The higher the inception score, the clearer and more diverse the generated images.

#### 4.1.2. Fréchet Inception Distance

The IS only considers the generated samples, and thus cannot explicitly reflect the distance between the real and generated data. Thus, we further adopted the Fréchet inception distance [46] to measure the divergence between our results and real photos. The lower the Fréchet inception distance, the more the distributions of real photos and our photorealistic rendering results overlap.

### 4.2. Comparison with Other State-of-the-Art Works

We compare the visual effects of face attribute transfer with other state-of-the-art methods in this section. The competitors include:

The neural algorithm of artistic style, proposed by Gatys et al. [3], is the pioneering work of neural style transfer. This method adopt the representations derived from convolutional neural networks (CNNs) to extract hight-level image information, and iteratively optimizes each input image to match the representations of a reference image. We call this work NST in the following for simplicity.

BN statistics matching, proposed by Li et al. [6], regards the neural style transfer as a special domain adaptation problem. To find evidence supporting this viewpoint, inspired by the observation that the batch normalization (BN) layer reflects the distributions of different domains, Ref. [6] designed a new style loss by aligning the BN statistics to replace the original Gram-matrix-based style loss [3], which requires less computation and can yield visually promising style transfer results.

WCT, proposed by Li et al. [7], can generalize to arbitrary unseen styles and attributes without needing to be explicitly trained on predefined styles. To achieve this, the method transforms the content features using classic whitening and coloring transforms (WCTs) with regard to the style features.

AvatarNet, proposed by Sheng et al. [8], renders multi-scale styles from an arbitrary reference in one feed-forward process by embedding a patch-based feature manipulation module called style decorator into a reconstruction network to fuse multi-scale style features, which shows much faster speed than WCT [7].

Deep photo style transfer, proposed by Luan et al. [11] to perform photographic style transfer, mitigates two main weaknesses of previous techniques: the painting-like effects and the content-mismatch problem. Similar to our approach, this work introduces guidance to the procedure of style transfer based on the semantic segmentation of both inputs and exemplars. We call this work DPST in the following for simplicity.

**Figure 5:**
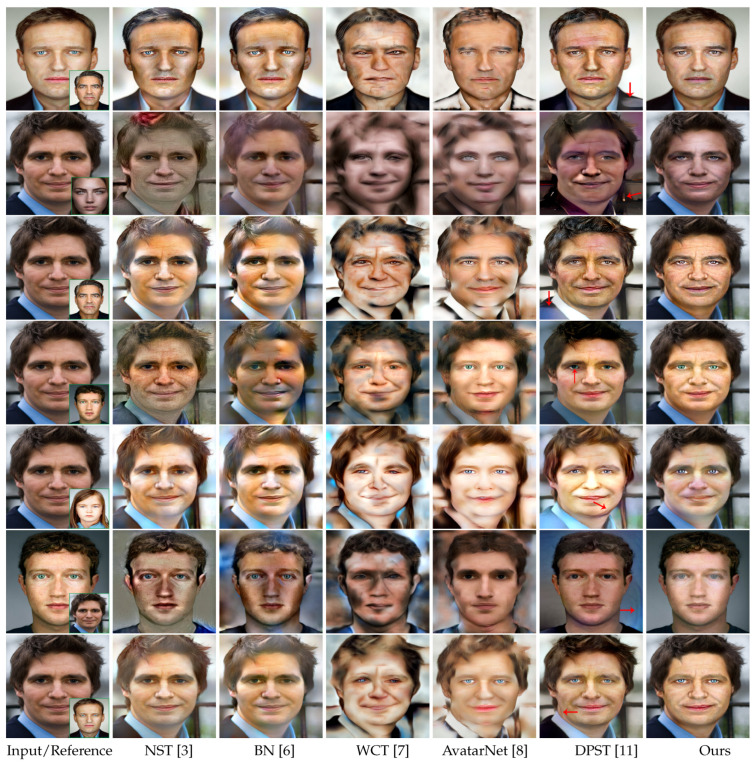
Visual comparison of face attribute transfer with other state-of-the-art methods.

For the competitors mentioned above, DPST is the only local method that uses the semantic map to constrain the transfer process to only occur between semantically identical regions. NST, BN, WCT, and AvatarNet are all global, so do not apply the semantic map concept. The codes of competitors we used were all provided by the authors. Figure 5 shows the visual results of the comparison of our proposed method (the regions except for the eyes, nose, mouth, and face skin are labeled as background) with other state-of-the-art methods. It can be observed that for the global methods, NST and BN lack the richness and diversity of the attribute pattern due to the greedy optimization they use, and their optimization procedures are unstable and prone to getting stuck in local minima. Though WCT significantly promotes the richness and faithfulness of the style and attribute, it tends to over-distort the image content and draws unseen patterns. Compared with WCT, AvatarNet is capable of producing more natural results. However, AvatarNet and other global competitors still suffer from some common limitations. In particular, in addition to causing painting-like effects (the transfer result looks like a painting instead of a photograph), these method fail to avoid undesirable transfers between semantically unrelated regions. That is, the global style transfer methods simply transfer the overall style from the reference to the whole input, instead of mapping the style of a local object to another semantically equivalent one (e.g., mouth-to-mouth and eyes-to-eyes), which can cause the attribute of an organ to spill over into the rest of the face photo. Though DPST is able to respect the semantics of objects, it is inferior to our method in terms of producing photographic outputs. Specifically, as indicated by the red arrows in the sixth column in Figure 5, some local structures in the results of DPST are distorted and some painting-like artifacts appear, which should not occur in the photos taken by users. In addition, all the global and local competitors fail to solely transfer the attributes of the face organs in the foreground, while strictly maintaining other attributes (the attributes of the background region) the same as the original input image. In comparison, our method provides advantages compared with the above-mentioned methods. Firstly, our framework considers the semantics of each face organ, and our results are more faithful to the attribute in the reference image, including the texture, color, and illumination. Secondly, compared with DPST, our method better produces photographic attribute transfer effects. Thirdly, only our method has the ability to only transfer the attributes of one or two face organs, making it superior to other methods.

To quantitatively verify the effectiveness of our framework, we further conducted experiments on the Helen face dataset [47], which contains 2330 face images and the corresponding manually annotated semantic maps. We processed the Helen face dataset with 5 extra headshot portraits from database provided by [2] as the references. That is, each compared model produced 11,650 images, on which the inception score and the Fréchet inception distance were computed. Table 1 shows that our framework outperformed its competitors in both of these metrics, demonstrating that our results are of better quality (clarity and diversity) and are closer to the distribution in the real photos.

### 4.3. Flexibility Verification

**Figure 6:**
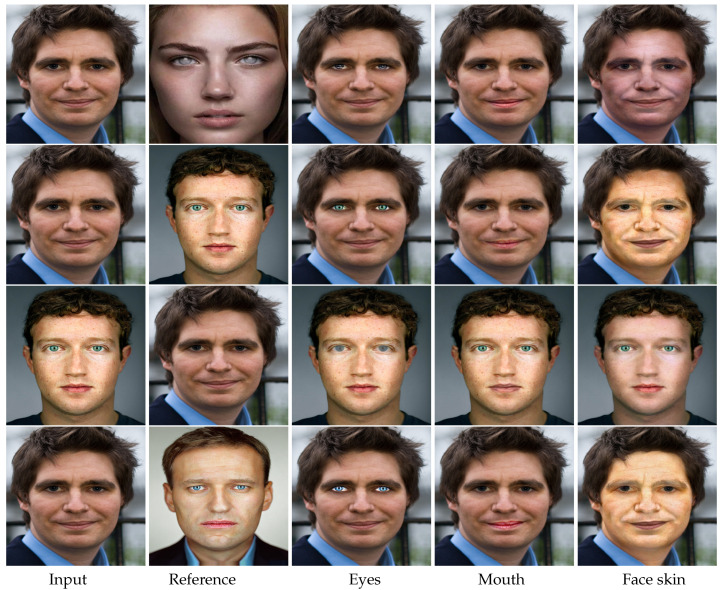
Additional visual results on separately transferring the attributes of eyes, mouth, and face skin from the same reference image.

To verify the flexibility of our framework, we further conducted two experiments. The first one was to only edit the attribute of a specific organ. We used the images in the first and second columns shown in Figure 6 as the input and reference, respectively. For transferring the attribute of a specific organ, we set the regions except this organ as the background in the semantic map. Taking editing the eyes as the attribute as an example, the regions except the eyes, including the mouth, face skin, hair, and other regions, were all labelled as background in the semantic map of both the input and reference images. Then, we processed the input image using our framework to produce the results of only changing the eyes attribute. The visual results produced by our method on separately transferring the attributes of eyes, mouth, and face skin from one reference image are shown in Figure 6. It can be seen that our framework is able to successfully alter the attribute of one specific face organ without affecting other organs, and allow users to flexibly determine which face organ to change and which to maintain by editing the semantic map.

**Figure 7:**
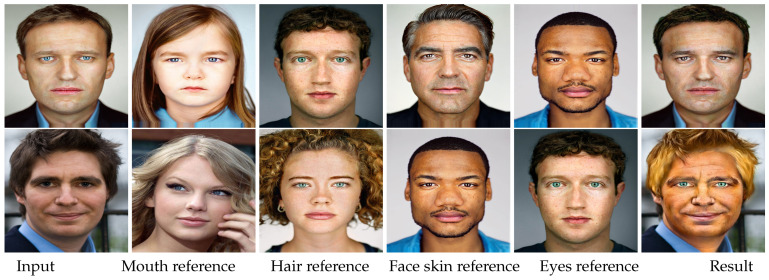
The results of transferring the facial attributes from multiple reference images to the same input images. The right-most column shows the results obtained by transferring the attributes of mouth, hair, face skin, and eyes from the 2nd column to the 5th column, respectively.

In addition, one may want to take two or more reference images and produce results simultaneously containing the attributes from these references in a controllable fashion. As the results of editing a specific organ can be viewed as the input images again, we can iteratively transfer the attribute of different organs from different reference images. Thus, our second experiment was to iteratively alter the attribute of different organs in the order of mouth, hair, face skin, and eyes from multiple reference images. Specifically, we first transferred the mouth attributes from the reference images shown in the second column of Figure 7 using the strategy adopted in the first experiment. Then, we transferred the attribute of hair from the reference images in the third column to the previously processed results. This procedure was repeated until the last attribute (eyes) was treated. The results are shown in Figure 7. As can be seen, by combining the attributes of multiple images, the visual results can be richer and more diverse.

### 4.4. Ablation Analysis

**Figure 8:**
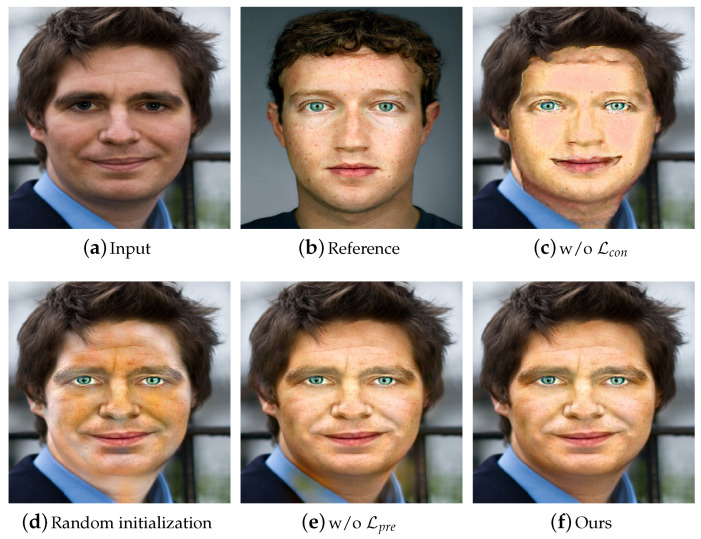
Effectiveness analysis of different strategies. (**c**,**e**) The results when neglecting $L_{con}$ and $L_{pre}$, respectively; (**d**) the result of randomly initializing the image by white noise.

We analyzed the effects of three different strategies: (1) the content term $L_{con}$ being omitted during training; (2) the image for neural style transfer being randomly initialized by white noise; (3) the preservation term $L_{pre}$ being omitted during training. As can be clearly observed in Figure 8, removing the content term $L_{con}$ cannot reasonably preserve the basic structure of the human face, whereas removing our newly proposed preservation term $L_{pre}$ leads to the attributes of the large organs spilling over into the rest of the image, e.g., the background regions around the neck in Figure 8e taking the attributes of face skin. Furthermore, we tested the effect of performing gradient descent on an image randomly initialized by white noise. The corresponding result is depicted in Figure 8d. It can be seen that directly updating a white noise image leads to the degradation of the faithfulness of the stylistic match.

## 5. Conclusions

In this paper, we proposed a framework to perform local face attribute transfer considering the semantic of each face organ, which locally transfers the face attribute from the reference headshot photo to the user’s own photo with the semantic map as guidance. The first step of our overall pipeline is to detect the facial landmark of both the input image and the reference image, then warp the reference image to match the pose, shape, position, and the expression of the input image using thin plate spline. To provide a more accurate initialized image for attribute transfer, we proposed a novel image fusion strategy to fuse the input image and the warped reference image. Finally, the fused result is taken as the initialized image for face attribute transfer and iteratively updated until the overall objective decreases to a certain range, or the iteration step reaches the maximum value set by the users. Our framework can flexibly handle the need for the attributes of face organs in the foreground to be changed while the rest is maintained the same as the original input image. We conducted extensive experiments to evaluate the performance of our framework, which revealed the efficacy of our design and its superiority over other state-of-the-art alternatives.

## Figures and Tables

**Table 1:** Quantitative comparison with the state-of-the-art methods. For the Fréchet inception distance, a lower value indicates better performance. For the inception score, the higher the better.

	Method	NST	BN	WCT	AvatarNet	DPST	Ours
LULC							
**IS**↑		3.10	3.02	3.16	3.19	3.76	3.81
**FID**↓		112.24	108.46	98.87	103.52	86.52	80.31

## Data Availability

MDPI Research Data Policies at the pubilcly available datasets of published papers [2] (link: https://people.csail.mit.edu/yichangshih/portrait_web/ accessed on 14 May 2021), and [47] (link: http://pages.cs.wisc.edu/~lizhang/projects/face-parsing/ accessed on 14 May 2021). The images used in this paper are all from the above pubilcly available datasets.
